# Association between prenatal alcohol exposure and children's facial shape: a prospective population-based cohort study

**DOI:** 10.1093/humrep/dead006

**Published:** 2023-02-16

**Authors:** X Liu, M Kayser, S A Kushner, H Tiemeier, F Rivadeneira, V W V Jaddoe, W J Niessen, E B Wolvius, G V Roshchupkin

**Affiliations:** Department of Radiology and Nuclear Medicine, Erasmus MC University Medical Center Rotterdam, Rotterdam, the Netherlands; Department of Oral and Maxillofacial Surgery, Erasmus MC University Medical Center Rotterdam, Rotterdam, the Netherlands; Department of Genetic Identification, Erasmus MC University Medical Center Rotterdam, Rotterdam, the Netherlands; Department of Psychiatry, Erasmus MC University Medical Center Rotterdam, Rotterdam, the Netherlands; Department of Social and Behavioral Science, Harvard T. H. Chan School of Public Health, Boston, MA, USA; Department of Internal Medicine, Erasmus MC University Medical Center, Rotterdam, the Netherlands; The Generation R Study Group, Erasmus MC University Medical Center Rotterdam, Rotterdam, the Netherlands; The Generation R Study Group, Erasmus MC University Medical Center Rotterdam, Rotterdam, the Netherlands; Department of Pediatrics, Erasmus MC University Medical Center Rotterdam, Rotterdam, the Netherlands; Department of Radiology and Nuclear Medicine, Erasmus MC University Medical Center Rotterdam, Rotterdam, the Netherlands; Faculty of Applied Sciences, Delft University of Technology, Delft, the Netherlands; Department of Oral and Maxillofacial Surgery, Erasmus MC University Medical Center Rotterdam, Rotterdam, the Netherlands; Department of Radiology and Nuclear Medicine, Erasmus MC University Medical Center Rotterdam, Rotterdam, the Netherlands; Department of Epidemiology, Erasmus MC University Medical Center Rotterdam, Rotterdam, the Netherlands

**Keywords:** child health, prenatal alcohol exposure, epidemiology, explainable artificial intelligence, 3D facial shape analysis

## Abstract

**STUDY QUESTION:**

Is there an association between low-to-moderate levels of prenatal alcohol exposure (PAE) and children’s facial shape?

**SUMMARY ANSWER:**

PAE before and during pregnancy, even at low level (<12 g of alcohol per week), was found associated with the facial shape of children, and these associations were found attenuated as children grow older.

**WHAT IS KNOWN ALREADY:**

High levels of PAE during pregnancy can have significant adverse associations with a child's health development resulting in recognizably abnormal facial development.

**STUDY DESIGN, SIZE, DURATION:**

This study was based on the Generation R Study, a prospective cohort from fetal life onwards with maternal and offspring data. We analyzed children 3-dimensional (3D) facial images taken at ages 9 (n = 3149) and 13 years (n = 2477) together with the data of maternal alcohol consumption.

**PARTICIPANTS/MATERIALS, SETTING, METHODS:**

We defined six levels of PAE based on the frequency and dose of alcohol consumption and defined three tiers based on the timing of alcohol exposure of the unborn child. For the image analysis, we used 3D graph convolutional networks for non-linear dimensionality reduction, which compressed the high-dimensional images into 200 traits representing facial morphology. These 200 traits were used for statistical analysis to search for associations with PAE. Finally, we generated heatmaps to display the facial phenotypes associated with PAE.

**MAIN RESULTS AND THE ROLE OF CHANCE:**

The results of the linear regression in the 9-year-old children survived correction for multiple testing with false discovery rate (FDR). In Tier 1 where we examined PAE only before pregnancy (exposed N = 278, unexposed N = 760), we found three traits survived FDR correction. The lowest FDR-*P* is 1.7e–05 (beta = 0.021, SE = 0.0040) in Trait #29; In Tier 2b where we examine any PAE during first trimester (exposed N = 756; unexposed N = 760), we found eight traits survived FDR correction. The lowest FDR-*P* is 9.0e−03 (beta = −0.013, SE = 0.0033) in Trait #139. Moreover, more statistically significant facial traits were found in higher levels of PAE. No FDR-significant results were found in the 13-year-old children. We map these significant traits back to the face, and found the most common detected facial phenotypes included turned-up nose tip, shortened nose, turned-out chin, and turned-in lower-eyelid-related regions.

**LIMITATIONS, REASONS FOR CAUTION:**

We had no data for alcohol consumption more than three months prior to pregnancy and thus do not know if maternal drinking had chronic effects. The self-reported questionnaire might not reflect accurate alcohol measurements because mothers may have denied their alcohol consumption.

**WIDER IMPLICATIONS OF THE FINDINGS:**

Our results imply that facial morphology, such as quantified by the approach we proposed here, can be used as a biomarker in further investigations. Furthermore, our study suggests that for women who are pregnant or want to become pregnant soon, should quit alcohol consumption several months before conception and completely during pregnancy to avoid adverse health outcomes in the offspring.

**STUDY FUNDING/COMPETING INTEREST(S):**

This work was supported by Erasmus Medical Centre, Rotterdam, the Erasmus University Rotterdam, and the Netherlands Organization for Health Research. V.W.V.J. reports receipt of funding from the Netherlands Organization for Health Research (ZonMw 90700303). W.J.N. is a founder, a scientific lead, and a shareholder of Quantib BV.

**TRIAL REGISTRATION NUMBER:**

N/A.

## Introduction

High levels of prenatal alcohol exposure (PAE) during pregnancy can have significant adverse effects on a child's health development resulting in fetal alcohol spectrum disorder (FASD). FASD is defined as a combination of growth retardation, neurological impairment and recognizably abnormal facial development (Jones and Smith, 1973; Poskitt 1984). The association of low–moderate PAE with the child’s health is less known, but could still have severe consequences for the child's health, including lower birth weight, smaller birth size, and preterm birth (Little, 1977; Jaddoe *et al.*, 2007).

Facial morphology can serve as a biomarker for health conditions and indicate developmental problems (Zaidel *et al.*, 2005; Smith *et al.*, 2006; Tzahor, 2009; Cordero *et al.*, 2011). Since low–moderate PAE is associated with the child’s development, it might be also associated with the facial morphology. However, the results of previous research are ambiguous.

Douglas and Mutsvangwa (2010) summarized the main approaches to detect the association between PAE and the human face and concluded that compared with direct craniofacial anthropometry (Farkas, 1994) or measurement from photographs (Astley and Clarren, 1996), 3-dimensional (3D) surface imaging is the most promising way to reduce measurement error. For this reason, Muggli *et al.* (2017) performed a regression using spatially-dense facial quasi-landmark coordinates as the outcome variables, in a moderately sized study of 434 12-month-old infants. After adjusting for potential covariates, significant facial trait associations were found at low–moderate levels of PAE, mainly with shape of the forehead, nose, and areas near eyes. Howe *et al.* (2019) performed a landmark-distance measurement on the 3D face in 4233 children (mean age: 15.4), but found no evidence of association with low–moderate PAE. There might be three reasons explaining why Muggli *et al.* (2017) and Howe *et al.* (2019) had opposite outcomes. (i) The association between low–moderate PAE and facial morphology of children does not exist; (ii) The association exists. However, the landmark-distance approach used in the Howe *et al.* (2019) study does not capture the complexity of facial morphology and thus missed the association; (iii) The association exists. However, the participants in the Howe *et al.* (2019) study were much older than those in the study of Muggli *et al.* (2017) and the association of PAE with the facial morphology might attenuate during childhood and adolescence. The present study clarifies this and explores which reason is the truth.

Recently, deep neural networks (DNNs) (LeCun *et al.*, 2015), a data-driven method, which can extract key information from high-dimensional input data, has become the state-of-the-art method for clinical applications (Miotto *et al.*, 2016; Asaoka *et al.*, 2020; Dwivedi *et al.*, 2020). One type of DNN architecture used for dimensionality reduction is the auto-encoder (Kingma and Welling, 2019) typically consisting of an encoder and decoder. The encoder is able to compress the high-dimensional 3D facial shape into low-dimensional representations of the facial morphology. The decoder then resamples these representations back to reconstruct the 3D facial shape.

In this article, we applied a deep learning algorithm to 3D photographs of children from a multi-ethnic prospective pregnancy cohort. We used an auto-encoder to reduce the facial complexity and then examined association of low-dimensional representation with PAE before and during pregnancy. Furthermore, we also attempted to predict PAE from children's facial shape, to test if facial morphology can be a biomarker that provides additional and independent clues for PAE diagnosis.

## Materials and methods

### Design and study population

**Figure 1.**
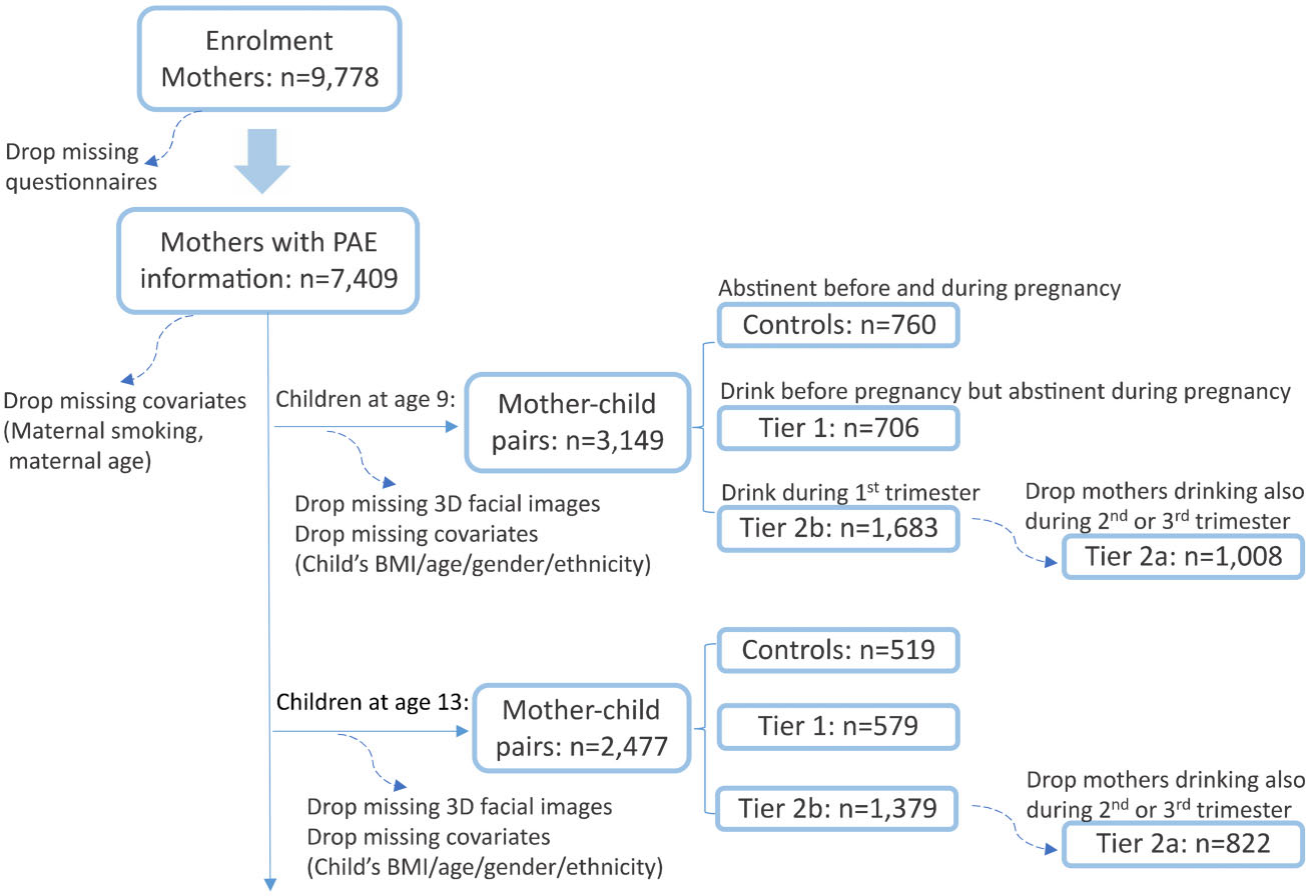
**Flow chart of data cleaning, and definition of Tier 1, Tier 2a, and Tier 2b.** In Tier 2, 99% of mothers drank before as well as during pregnancy. PAE: prenatal alcohol exposure; 3D: 3-dimensional.

This study was embedded in the Generation R Study, an ongoing population-based cohort study of pregnant women and their children from fetal life onwards. The goal is to identify early environmental and genetic causes leading to normal and abnormal growth, development, and health (Jaddoe *et al.*, 2006). All women living in the study area of Rotterdam, the Netherlands, who delivered between April 2002 and January 2006 were eligible. A total of 9778 participants were enrolled in the study. Information about maternal alcohol consumption was obtained by postal questionnaires in early, mid-, and late pregnancy. Response rates for these questionnaires were 91%, 80%, and 77% respectively (Jaddoe *et al.*, 2007). After removing missing questionnaires, 7409 (76%) mothers have completed alcohol information before and during pregnancy. 3D facial images of the children were taken at 9 and 13 years old. The population of children consisted of 17 different ethnicities. We selected four major ethnicities (Dutch, non-Dutch Western, Turkish, and Moroccan) and clustered them into two groups: Western (including Dutch and non-Dutch Western) and non-Western (including Turkish and Moroccan). After data cleaning, 3149 9-year-old children and 2477 13-year-old children were included in our study, with 1878 children assessed at both ages. A flow chart in Fig. 1 shows the data cleaning process and division of the population.

### Details of ethics approval

The study was approved by the Medical Ethics Committee (MEC) of the Erasmus Medical Center Rotterdam, the Netherlands (MEC 198.782/2001/31), and written informed consent was obtained from all participants themselves, or on that of their guardians/parents.

### Alcohol measurements

Mothers who reported any drinking were asked to classify their average alcohol consumption into one of the following six levels: <1 drink per week; 1–3 per week; 4–6 per week; 1 per day; 2–3 per day; and >3 per day. An average alcoholic drink contains about 12 g of alcohol. The subject number of each level could be found in Supplementary Table SI.

We defined three tiers to understand the association of PAE in different pregnancy stages. In all tiers, mothers who were abstinent before and during pregnancy comprised the control group. Settings of the exposed groups are shown in Fig. 1. Tier 1 defined mothers only drinking up to 3 months before pregnancy as the exposed group, while Tier 2 defined mothers drinking during pregnancy as the exposed group. In Tier 2a, mothers who drank during the first trimester of pregnancy but were abstinent during the other trimesters constituted the exposed group. Tier 2b followed similar exposure definitions as Tier 2a but included mothers who also drank during the other trimesters in the exposed group. It is worth noting that 99% of mothers who drank during pregnancy also drank up to 3 months before pregnancy.

### Data preprocessing

The 3D face images were collected with the 3dMD cameras system (3dMD Corp). The distance and angle between the participants and cameras were fixed when taking photos. We adopted 3D morphology registration pipelines (Booth *et al.*, 2018) to build the raw data into a template-based dataset, in which each facial shape was modeled by a 3D graph (Hsu and Jain, 2001) with the same vertex number and edge connectivity. Details about the data preprocessing can be found in the Supplementary Material.

### Dimensionality reduction to generate facial traits

**Figure 2.**
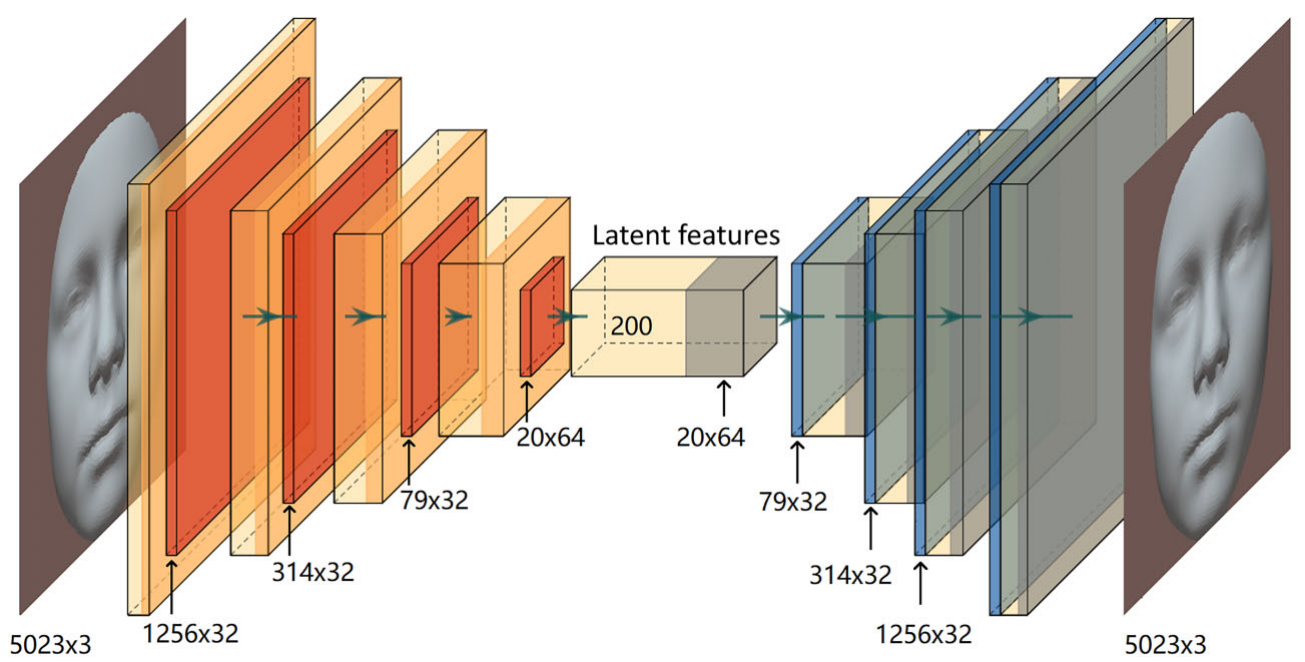
**Framework of the auto-encoder.** Red: down-sample process via four encoding layers; Blue: up-sample process via four decoding layers. The size of the input face is 5023 × 3, which means it contains 5023 vertices and each vertex has three features (*x*, *y*, and *z* coordinates). In down-sample process, the size of the input face is gradually reduced into 1256 × 32 (1256 vertices and 32 features), 314 × 32, 79 × 32, 20 × 64, and 200 (200 latent facial traits). The up-sample process is mirrored from the down-sample process.

We used a 3D graph auto-encoder (Gong *et al.*, 2019) for high-dimensional facial shape analysis. As shown in Fig. 2, the auto-encoder consists of encoder and decoder that can perform feature mapping in a non-linear manner. The encoder compresses the high-dimensional 3D facial shape into N latent features, while the decoder reconstructs the 3D facial shape from the latent features. By minimizing the error between input and reconstructed facial shapes (Supplementary Fig. S1), the main facial morphology is captured in the N latent features. The encoding and decoding process can be formulated as:


(1) $$Z=\mathrm{Encode}\left(F\right),$$

(2) $$F^{'}=\mathrm{Decode}\left(Z\right),$$

where Z = $\left[z_{0},z_{1},\ldots ,z_{N}\right]$ refers to the N latent features, Encode() and Decode() refer to the down-sampling and up-sampling process respectively. F denotes the input 3D facial shape, while F′ represents the reconstructed facial shape. In this article, we defined these N latent features as N facial traits. In order to make a tradeoff between reconstruction error and dimensional complexity, we conducted experiments on different numbers of traits (Supplementary Fig. S2). The optimum number was found to be 200. As shown in Supplementary Fig. S3, each trait represents different facial phenotypes. Supplementary Fig. S8 shows the correlations between these 200 traits. A uniform measurement f(z) was defined in Supplementary Equation S1, to measure the effect size of each trait on the facial shape. More implementation details can be found in the Supplementary materials.

### Statistical analysis

After the dimensionality reduction each 3D facial image is represented by 200 traits. We performed linear regression analysis where each trait was entered as dependent variable. We ran independent linear regression models for 200 facial traits. To correct the p-value for multiple testing, we calculated the false discovery rate (FDR) with **α = **0.05. We selected FDR-significant traits and mapped them back to the 3D facial shape to visualize facial features linked with PAE. Supplementary Figs. S3 and S4 explain implementation details about the mapping. To partially demonstrate that the extracted phenotypes can represent known biologically-driven differences among populations, we tested the proposed pipeline on visualizing the facial features linked with sex (Supplementary Table SVI and Supplementary Fig. S7). The result is in line with previous studies (Matthews *et al.*, 2018; Zhang *et al.*, 2022). The statistical analysis we used in this study is based on a trait-by-trait univariate approach. However, multivariate approaches such as partial least squares regression and canonical correlation analysis could be alternative solutions. We compared our univariate approach with multivariate approaches (Supplementary Table SVIII and Supplementary Fig. S12). No obvious difference between our univariate approach and multivariate approaches was found in this study. Since multivariate approaches might have the problem of overfitting (Faber and Rajkó, 2007), we adopted the trait-by-trait univariate approach through this study for more conservative results.

**Figure 3.**
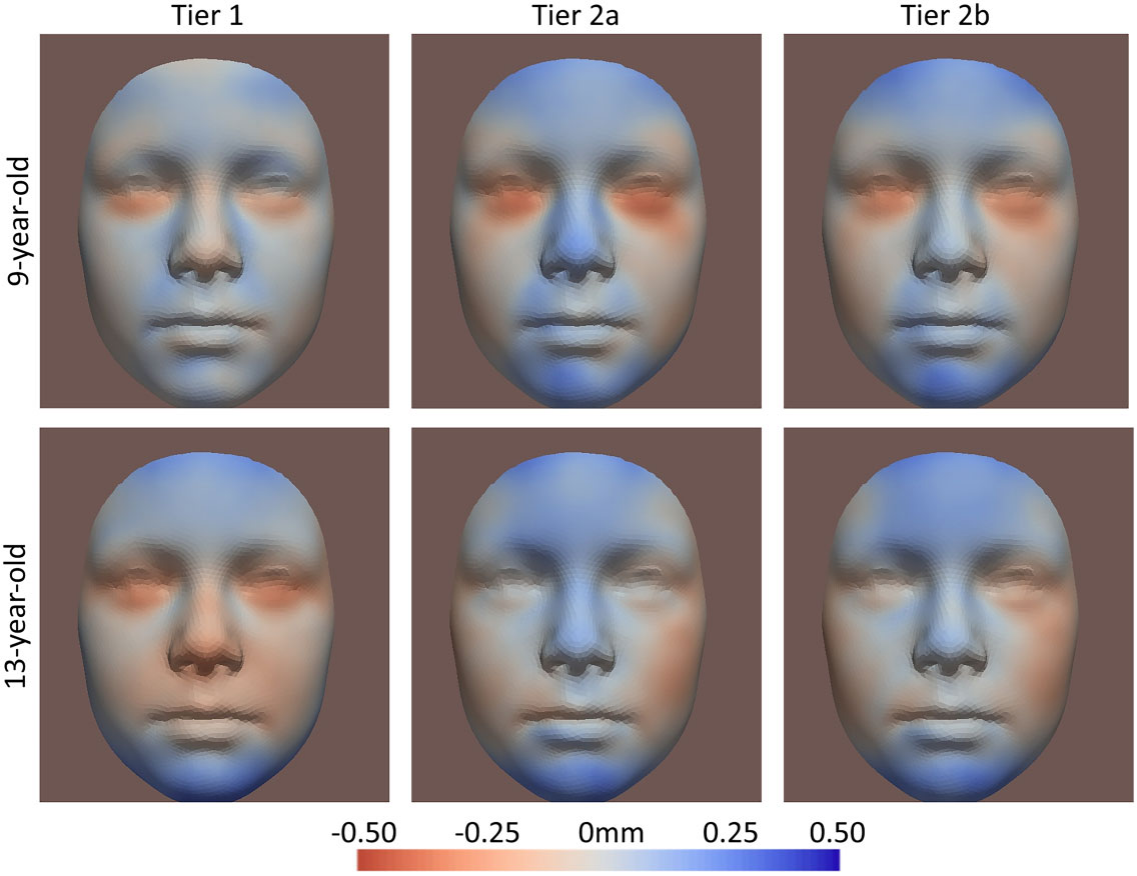
**Nominal significant results of different tiers.** Multi-ethnic, prenatal alcohol exposure (PAE) level >1. Tier 1: PAE only before pregnancy; Tier 2a: PAE during first trimester, but abstinent during trimesters two and three; and Tier 2b: PAE during first trimester, or PAE during all trimesters. Red areas refer to inward changes while blue areas refer to outward changes of the face with respect to the geometric center of the head.

**Figure 4.**
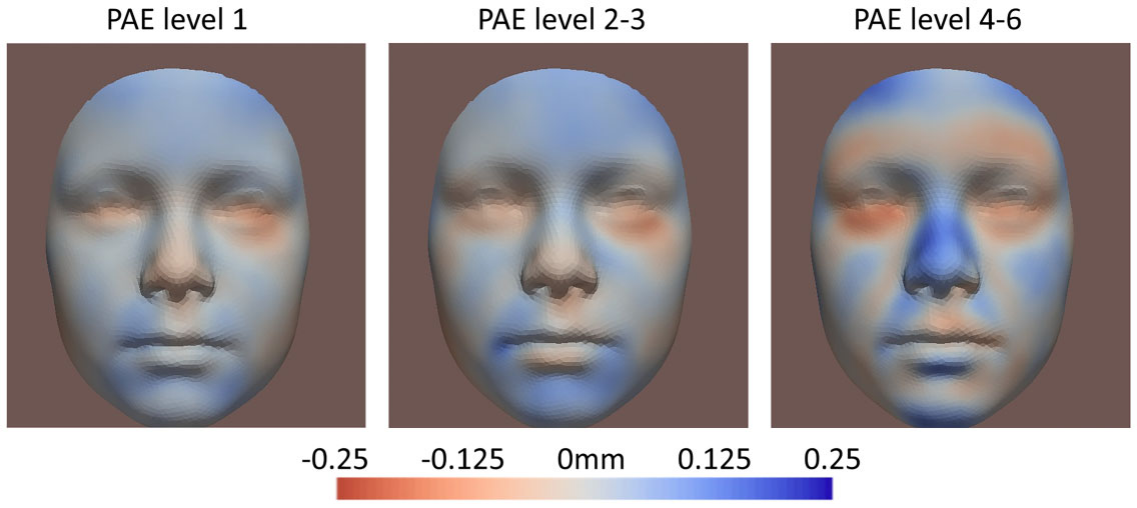
**FDR (false discovery rate)-significant results of different levels of prenatal alcohol exposure (PAE), in Tier 2b, multi-ethnic group for 9-year-old children.** Level 1: <12 g of alcohol per week, N = 887; Levels 2 and 3: 12–72 g per week, N = 546; Levels 4–6: >12 g per day, N = 79. Mothers who ever drank heavily (>72 g/day) were excluded in the dose–response assessment. Red areas refer to inward changes while blue areas refer to outward changes of the face with respect to the geometric center of the head.

**Figure 5.**
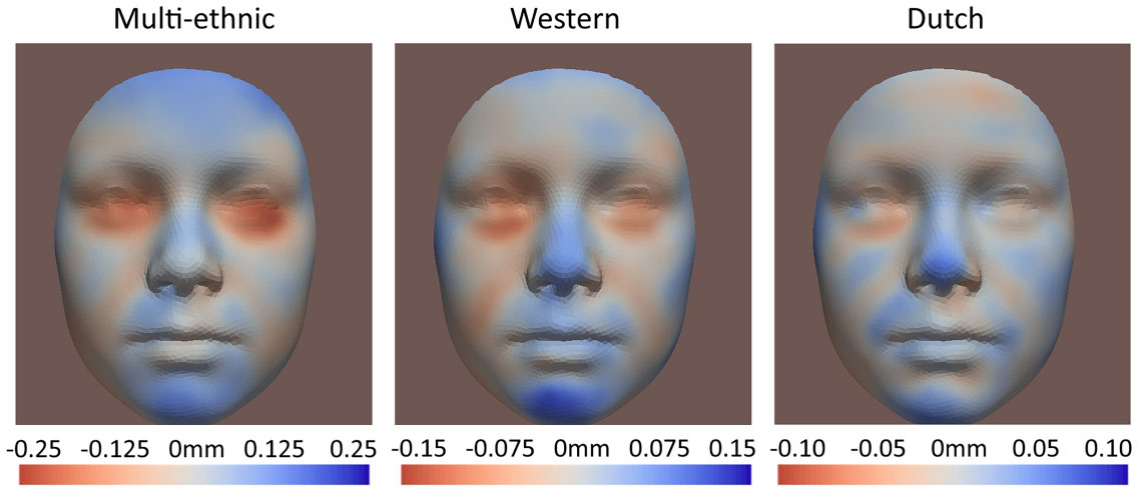
**FDR (false discovery rate)-significant results of the multi-ethnic (Dutch, non-Dutch Western, Turkish, and Moroccan), the Western (Dutch and non-Dutch Western) and the Dutch-only samples, PAE (prenatal alcohol exposure) level >1 for 9-year-old children in Tier 2b (any PAE during first trimester).** Red areas refer to inward changes while blue areas refer to outward changes of the face with respect to the geometric center of the head.

The linear regression analysis was stratified for four factors: tier of exposure (Table II and Fig. 3), level of exposure (Supplementary Table SII, Fig. 4, and Supplementary Fig. S6), child ethnicity (Fig. 5), and age (Supplementary Fig. S5). In the stratification for ethnicity, we additionally performed a Dutch-only analysis (Supplementary Table SIX), because most subjects (>86%) in the exposed group are Dutch, while statistical adjustment for ethnicity via linear regression is not as good as a direct stratification. As for the stratification of age, the analysis was performed separately in the 9-year-old children, the 13-year-old children, and defined ‘growth’. The ‘growth’ was defined as the trait differences between the 9- and 13-year-old children, including children assessed twice. The traits of ‘growth’ were computed by:


(3) $$Z_{\mathrm{growth}}=\;Z_{13}-\;Z_{9},$$

where $Z_{\mathrm{growth}}\;$refers to the growth of traits,$\;Z_{13}$ and $Z_{9}$ are traits of 13-year-old and 9-year-old children, respectively.

For all stratifications, the regression covariates included potential confounders: ethnicity, maternal age, maternal smoking in pregnancy, children BMI, age, and gender, where the ethnicity was coded as a dummy variable.

### Phenotypes recognition for PAE

The PAE prediction was only performed in children of Dutch national origin. We used logistic regression to model binary prediction, where 200 facial traits were used for prediction of PAE. Non-exposed children were set as the control group, while children with PAE level >1 were set as exposed group. The prediction accuracy was quantified with the area under the receiver operating characteristic curve (AUC), with 5-fold cross-validation. To adjust for potential biases, we first set the above-mentioned covariates as independent variables and obtained the baseline results (Model A). Then, we added facial traits as independent variables to the baseline model (Model B).

*F*-test is often used to identify the model that best fits the population from which the data were sampled (Ludden *et al.*, 1994). Here, we performed ANOVA *F*-tests separately on Model A and Model B and then determined the *F*-value as well as *P*-value for each independent variable.

## Results

The characteristics of the study population are summarized in Table I. Maternal smoking, maternal age, child BMI, and especially ethnicity showed imbalanced distribution between the control and exposed groups. The Dutch group had the highest proportion of different characteristics, accounting for about 45% of the control group, while above 86% of the exposed groups. Supplementary Table SI further shows details about the number of children of each level of PAE.

**Table I:** Characteristics of children and their mothers included in the analysis.

Characteristic	**Control** **(Abstinent)**	**PAE Tier 1** **(Exposed)**	**PAE Tier 2a** **(Exposed)**	**PAE Tier 2b** **(Exposed)**
**9-Year-old children (N = 3149)**				
**In total**	N = 760	N = 706	N = 1008	N = 1683
**Ethnicity (%)**				
** Western: Dutch**	328(43.2)	608(86.1)	872(86.5)	1476(87.7)
** Western: Non-Dutch**	39(5.1)	74(10.5)	119(11.8)	181(10.8)
** Non-Western: Turkish**	212(27.9)	20(2.8)	7(0.7)	16(1.0)
** Non-Western: Moroccan**	181(23.8)	4(0.6)	10(1.0)	10(0.6)
**Child's gender, No. (%)**				
** Male**	357(47.0)	306(43.3)	489(48.5)	823(48.9)
** Female**	403(53.0)	400(56.7)	519(51.5)	860(51.1)
**Child BMI, mean (SD)**	18.6(3.2)	17.4(2.6)	17.1(2.2)	17.0(2.1)
**Child age, mean (SD), years**	9.8(0.4)	9.7(0.3)	9.8(0.3)	9.8(0.3)
**Maternal smoking, No. (%)**				
** No**	556(73.2)	406(57.5)	508(50.4)	846(50.3)
** Yes**	204(26.8)	300(42.5)	500(49.6)	837(49.7)
**Maternal age, mean (SD)**	28.2(5.0)	30.4(4.7)	31.2(4.3)	31.7(4.1)
**13-Year-old children (N = 2477)**				
**In total**	N = 519	N = 579	N = 822	N = 1379
**Ethnicity (%)**				
** Western: Dutch**	237(45.7)	502(86.7)	714(86.9)	1206(87.5)
** Western: Non-Dutch**	33 (6.4)	61(10.5)	95(11.6)	152(11.0)
** Non-Western: Turkish**	122(23.5)	11(1.9)	10(1.2)	17(1.2)
** Non-Western: Moroccan**	127(24.5)	5(0.9)	3(0.4)	4(0.3)
**Child's gender, No. (%)**				
** Male**	247(47.6)	263(45.4)	407(49.5)	690 (50.0)
** Female**	272(52.4)	316(54.6)	415(50.5)	689 (50.0)
**Child BMI, mean (SD)**	21.1(4.0)	19.6(3.3)	19.1(2.7)	19.0(2.6)
**Child age, mean (SD), years**	13.7(0.4)	13.55(0.3)	13.6(0.3)	13.6(0.3)
**Maternal smoking, No. (%)**				
** No**	372(71.7)	346(59.8)	417(50.7)	708 (51.3)
** Yes**	147(28.3)	233(40.2)	405(49.3)	671 (48.7)
**Maternal age, mean (SD)**	28.2(5.1)	30.7(4.7)	31.2(4.2)	31.7 (4.1)

PAE: prenatal alcohol exposure; Tier 1: PAE only before pregnancy; Tier 2a: PAE during first trimester, but abstinent during the other trimesters; Tier 2b: PAE during first trimester, or PAE during all trimesters.

The results of the linear regression in the 9-year-old children survived correction for multiple testing with FDR. PAE before pregnancy (Tier 1) and during pregnancy (Tiers 2a and 2b) was associated with facial traits (Table II), and more FDR-significant facial traits were found in Tier 2 than in Tier 1. Supplementary Table SII further shows results of dose–response assessment for different levels of PAE: more FDR-significant facial traits were found in higher levels of PAE.

No FDR-significant results were found in the 13-year-old children, or for the ‘growth’ in the longitudinal analysis. The nominal significant results (*P*-value <0.05) for 9-year-old, 13-year-old, and ‘growth’ can be found in Supplementary Table SIII.

Each facial trait (index 0–199) represents different facial phenotypes, which can be found in Supplementary Fig. S3.

### Visualization of results

Figure 5 shows the facial shape transformations from control to the Tier 2b group for the multi-ethnic, Western, and Dutch-only samples at 9 years old. Figure 4 shows the shape transformations in the multi-ethnic samples in Tier 2b at 9 years old, stratified for different PAE levels. Figure 3 shows the shape transformations for different tiers. Supplementary Figure S5 shows the shape transformations for different ages (9 years old, 13 years old, and the ‘growth’). Figures 4 and 5 are based on FDR-significant results, while Fig. 3 and Supplementary Fig. S5 are based on nominal significant (*P*-value <0.05) results. For all heatmaps, red areas refer to inward changes while blue areas refer to outward changes of the face with respect to the geometric center of the head (Supplementary Fig. S3). Supplementary Fig. S4 explains how each heatmap was generated, by combining represented phenotypes of each significant trait using their coefficients as weights. The most common detected facial phenotypes included turned-up nose tip (mostly contributed by Traits #36 and #69), shortened nose (#51 and #87), turned-out chin (#51 and #57), and turned-in lower-eyelid-related regions (#51, #69#, and 57#).

### Phenotypes recognition for PAE

Table III shows the prediction results of the logistic regression, where Model B with facial traits as independent variables obtained slightly higher AUC than Model A (baseline). Supplementary Table SIV further shows the odds ratios (OR) of traits in the logistic regression. The highest OR is 1.25 (*P* = 0.008; trait index 36) and 1.36 (*P* = 0.004; trait index 14) for 9- and 13-year-old children, respectively. Supplementary Table SV shows the ANOVA *F*-test results, which confirmed that the prediction model was improved when facial traits were included.

**Table II:** Details about FDR-significant traits in the multi-ethnic group.

Trait index	*P*-value	FDR-corrected *P*-value	Coefficient	Standard error	Mean	Standard deviation	*f*(*z*)
**Tier 1 (PAE only before pregnancy): Ne = 278, Nc = 760**							
**29 (W) (D)**	8.3e−08	1.7e−05	0.021	0.0040	0.0079	0.052	197.95
**44 (W) (D)**	1.8e−04	2.0e−02	−0.014	0.0038	0.0068	0.048	165.52
**173**	4.2e−04	3.0e−02	−0.014	0.0039	−0.0060	0.050	185.23
**Tier 2a (PAE during first trimester, but abstinent during the other trimesters). Ne = 563, Nc = 760;**							
**36 (W) (D)**	4.2e−06	8.5e−04	0.021	0.0046	0.0105	0.064	269.78
**29**	3.8e−05	3.8e−03	0.014	0.0035	0.0079	0.052	197.95
**139 (W)**	1.0e−04	6.9e−03	−0.014	0.0035	0.0065	0.048	189.49
**173**	2.8e−04	1.4e−02	−0.013	0.0035	−0.0060	0.050	185.23
**51**	5.7e−04	2.3e−02	−0.030	0.0087	−0.0016	0.125	833.34
**87 (W) (D)**	6.2e−04	2.1e−02	−0.011	0.0032	3.5e−04	0.044	155.85
**69**	9.5e−04	2.7e−02	0.019	0.0059	−0.0170	0.080	485.34
**125**	1.5e−03	3.8e−02	0.015	0.0047	0.0155	0.066	287.89
**Tier 2 b (any PAE during first trimester, or PAE during all trimesters): Ne = 756, Nc = 760**							
**36 (W) (D)**	7.1e−05	1.4e−02	0.017	0.0044	0.0105	0.064	269.78
**139(W)**	9.3e−05	9.3e−03	−0.013	0.0033	0.0065	0.048	189.49
**29**	1.9e−04	1.3e−02	0.013	0.0034	0.0079	0.052	197.95
**51**	2.5e−04	1.2e−02	−0.030	0.0083	-0.0016	0.125	833.34
**69**	5.6e-04	2.3e-02	0.019	0.0055	-0.0170	0.080	485.34
**173**	8.9e−04	3.0e−02	−0.011	0.0033	−0.0060	0.050	185.23
**87 (W) (D)**	1.3e−03	3.6e−02	−0.010	0.0031	3.5e−04	0.044	155.85
**57(W)**	1.9e−03	4.8e−02	0.022	0.0070	−0.0127	0.101	648.42

9-Year-old children for PAE level >1.

FDR: false discovery rate; PAE: prenatal alcohol exposure; Tier 1: PAE only before pregnancy; Tier 2a: PAE during first trimester, but abstinent during the other trimesters; Tier 2b: PAE during first trimester, or PAE during all trimesters.

Ne refers to the number of the exposed samples, while Nc refers to the number of the control samples.

As defined in Supplementary Equation (S1), *f*(*z*) is the effect size of the trait on the facial shape.

Facial trait index with ‘(W)’ means they are also significant in the Western-only samples.

Facial trait index with ‘(D)’ means they are also significant in the Dutch-only samples.

**Table III:** PAE prediction AUC with 5-fold cross-validation, for children of Dutch national origin, PAE level > 1, Tier 2b.

Models	Independent variables	**AUC in 9-year-old Ne = 670, Nc = 329**	**AUC in 13-year-old Ne = 543, Nc = 236**
Model A	Maternal age, maternal smoking in pregnancy, children BMI, age and gender	0.769	0.756
Model B	All independent variable in Model A, and 200 facial traits	0.782	0.757

PAE: prenatal alcohol exposure; Tier 2b: PAE during first trimester, or PAE during all trimesters; PAE level 1: <1 drink per week; PAE level 2: 1–3 per week; PAE level 3: 4–6 per week; PAE level 4: 1 per day; PAE level 5: 2 and 3 per day; PAE level 6: >3 per day.

An average alcoholic drink contains about 12 g of alcohol.

Ne refers to the number of the exposed samples, while Nc refers to the number of the control samples.

## Discussion

This study examined the association between PAE and children’s facial shape by performing a multi-ethnic population-based analysis, using state-of-the-art image analysis methodology including deep-learning approaches. A significant association between PAE and facial morphology was found in the 9-year-old children, with a dose–response relationship: more statistically significant facial traits were found in higher levels of PAE. The most common detected facial phenotypes included turned-up nose tip, shortened nose, turned-out chin, and turned-in lower-eyelid-related regions.

The association between low levels of PAE and children’s facial shape has been reported previously, but our study found an association at a much lower dose of exposure. Muggli *et al.* (2017) found a significant association at a low PAE level, <70 g of alcohol per week, in 12-month-old babies. Iveli *et al.* (2007) considered a light consumption <700 mL per trimester (roughly <46 g per week) and found 66% of 79 newborns in the exposed group had some facial abnormality. However, in Tier 2b which assessed the dose–response (Fig. 4 and Supplementary Table SII), where mothers who ever drank heavily (>72 g a day) were excluded, we found that even if mothers drank very little (<12 g per week) during pregnancy, the association between PAE and children’s facial shape could be observed.

The associations of PAE with children’s facial shape attenuated as children became older. As shown in Supplementary Fig. S5, the PAE-related patterns decreased from the ‘9-year-old’ column to the ‘13-year-old’ column. In addition, by accessing longitudinal data, we identified PAE-related growth patterns (‘growth’ is facial changes from 9 to 13 years defined as Equation (3)) which are consistent with the attenuation of PAE-related patterns from 9 to 13 years. The result of this longitudinal analysis is another piece of evidence that supports the attenuation of the association. The results of Muggli *et al.* (2017) and ours differ from those of Howe *et al.* (2019), where no association was found in the 15-year-old children. Possibly this discrepancy can be explained by a further attenuation of the association. Our results suggest that as children grow up, the association of PAE with children’s facial shape could attenuate. This finding is consistent with the associations of PAE with children’s weight, height, and head circumference that attenuate as children become older (Day *et al.*, 1999, 2002; Carter *et al.*, 2013). One possible explanation for this change over lifetime might be the impact of the environment. With age some alcohol-related phenotypes on the facial shape of children might be obscured by environmental influences, especially at the peak age (12–14 years for boys and 10–12 years for girls) of facial development (Baughan *et al.*, 1979; Mellion *et al.*, 2013). Further investigations on the mechanism of association are needed to fully understand how the association develops and then attenuates with age.

We compared our findings between different tiers and found that results were similar in Tiers 2a and 2b (Tier 2a: PAE during first trimester, but abstinent during the other trimesters; Tier 2b: any PAE during first trimester). This suggests that the associations were mainly explained by PAE during the first trimester of the pregnancy. Besides, we also examined the association of PAE before pregnancy with children’s facial shape. A significant association was found in Tier 1 (PAE only before pregnancy), but with less statistically significant facial traits than those in Tier 2 (PAE during pregnancy). It is worth noting that 99% of drinking mothers in Tier 2 also drank before pregnancy, but none of mothers drinking in Tier 1 also drank during pregnancy. Thus, the comparison (Table II and Fig. 3) between Tiers 1 and 2 indicated that more statistically significant facial traits were found associated with PAE in mothers who continued to drink during pregnancy (Tier 2), than in those who stopped when becoming pregnant (Tier 1). To the best of our knowledge, this study is the first to examine the association between PAE and children’s faces including exposures up to 3 months before pregnancy. Previous studies show that PAE before pregnancy is associated with other aspects of child developments, and the association is explained by maternal metabolic disorders such as impaired maternal glucose homeostasis and hepatic steatosis (Nykjaer *et al.*, 2014; Lee *et al.*, 2020; McDonald and Watson, 2020). The mechanism of the association with the face could be similar, but further investigations are needed to test this.

We performed an additional Dutch-only analysis and compared it with the multi-ethnic analysis. The top traits from Dutch-only result are consistent with the top traits from multi-ethnic result, but with overall higher *P*-values (Supplementary Table SIX). Moreover, the facial heatmaps are also consistent between Dutch-only and multi-ethnic results (Fig. 5 and Supplementary Fig. S5). It means that the linear regression model successful adjusted for ethnicity as confounding bias in the multi-ethnic group. Therefore, to increase the power, the multi-ethnic samples were included in all other stratifications.

### Strengths and limitations

This study is a well-described population-based prospective cohort of multi-ethnic children. We have a large sample size with detailed PAE data that allows exposure classifications not available in many other studies. For image analysis, the presence of non-linearity in the input 3D facial data has been a challenge for traditional approaches (e.g. principal component analysis, PCA) which can only capture the linearity in the input data (Ranjan *et al.*, 2018). To overcome this difficulty, we utilized a deep-learning approach, which enables non-linear mapping between the input 3D facial data and the latent traits (Gong *et al.*, 2019; Hanocka *et al.*, 2019). In our additional experiments (Supplementary Table SX), the improvement of the deep-learning model was confirmed by improved generalization and specificity (Nauwelaers *et al.*, 2021) compared with the PCA-based model. Lastly, to better interpret and validate the results in a conventional manner, we integrated the deep-learning approach with traditional linear and logistic regression models. Benefiting from these settings, the method used in this study is sensitive enough to detect association with mild alcohol consumption.

To test the replicability of the deep-learning approach, we performed three additional independent trainings of the auto-encoder, and followed the linear regression analysis. Across these independent runs, Supplementary Table SVII shows similar indices of facial traits that survived the FDR. Moreover, Supplementary Fig. S11 shows similar visualization results on facial heatmaps. This means that our results are consistent and robust across independent runs. We also tested the correlation between latent traits (Supplementary Figs. S8, S9, and S10). The distribution of correlations between 200 latent dimensions is close to a Gaussian distribution with mean of 0.001 ± 0.078 (SD), which means that 95% of correlations are within 0.14 and 99.7% of correlations are within 0.24. Besides, for traits which were found statistically significant (in Table II and Supplementary Table SII), the highest correlation is 0.22. From this perspective, the correlations between the found significant traits are weak.

We also used derived traits for the prediction of PAE. Although the AUC increase with facial traits was rather small, the result still indicated that facial phenotypes provided additional and independent clues for association of PAE, which were confirmed by the OR and *F*-tests (Supplementary Tables SIV and SV).

Craniofacial development closely corresponds to brain development (Naqvi *et al.*, 2021). Thus, classic facial features of FASD such as short palpebral fissure, smooth philtrum, and thin upper lip have been linked to brain abnormalities and cognitive outcome in FASD and have been used to diagnose children at risk of developing neurobehavioral deficits (Suttie *et al.*, 2013; Smith *et al.*, 2014; Hoyme *et al.*, 2016; Muggli *et al.*, 2017). Low–moderate PAE has also shown adverse associations with children’s brain cognitive development, resulting in psychological and behavioral problems (Larroque *et al.*, 1995; Willford *et al.*, 2006; Lewis *et al.*, 2012; Lees *et al.*, 2020). Although the connection between these cognitive problems and facial phenotypes is still unknown, the traits we discovered in this study are potentially useful in identification of children at risk of developing these cognitive problems, which should be further substantiated in future studies.

We had no data for alcohol consumption more than three months prior to pregnancy and thus do not know if maternal drinking more than three months prior to pregnancy could also have effects or not. The self-reported questionnaire might not reflect the accuracy alcohol measurements because mothers may have denied their alcohol consumption.

## Conclusions

The results of this study suggest that low–moderate maternal alcohol consumption up to three months before and during pregnancy is associated with the facial appearance of children. The association with facial morphology of the offspring was attenuated with increasing age. Our results imply that facial morphology, such as quantified by the approach we proposed here, can be used as a biomarker in further investigations. Furthermore, our study suggests that women who are pregnant or want to become pregnant soon should quit alcohol consumption several months before conception and completely during pregnancy to avoid adverse health outcomes in the offspring.

## Supplementary Material

dead006_Supplementary_Data

## Data Availability

The data underlying this article are available in the article and in its supplementary material.
