# Supplementary material for: Association between prenatal alcohol exposure and children's facial shape: a prospective population-based cohort study
Source: Hum Reprod. 2023 Feb 16;38(5):961–72. doi: 10.1093/humrep/dead006 (PMC10152169; doi:10.1093/humrep/dead006)
Supplement: dead006_Supplementary_Data [file dead006_supplementary_data.docx]

**Association between prenatal alcohol exposure and children's facial shape. A prospective population-based cohort study**

X. Liu^1,2^, M. Kayser^3^, S. A. Kushner^4^, H. Tiemeier^5^, F. Rivadeneira^6,7^, V. W. V. Jaddoe^7,8^, W. J. Niessen^1,9^, E.B. Wolvius^2^, G. V. Roshchupkin^1,10*^

**Supplementary**

| **Content** | **Page number** |
| --- | --- |
| 1. Details about data pre-processing | 2 |
| 1. Implementation details about 3D graph convolutional networks | 3 |
| 1. Details about the study population of different levels of prenatal alcohol exposure (PAE) | 7 |
| 1. Details about dose-response assessment | 8 |
| 1. Details about nominal significant traits | 9 |
| 1. Detailed visualization results | 11 |
| 1. Detailed PAE prediction results | 12 |
| 1. Visualize the difference between sex groups | 13 |
| 1. Correlations between latent dimensions | 14 |
| 1. Replicability of the auto-encoder | 15 |
| 1. A test of the Multivariate approach | 16 |
| 1. Dutch-only Analysis | 17 |
| 1. Comparison between autoencoder (AE) and principal component analysis (PCA) | 18 |

**'**

Abbreviations:

PAE: prenatal alcohol exposure;

FDR: false discovery rate;

Tier 1: PAE only before pregnancy;

Tier 2a: PAE during first trimester, but abstinent during the other trimesters;

Tier 2b: PAE during first trimester, or PAE during all trimesters;

3D: 3-dimensional

**1. Details about data pre-processing**

1. **Data collection:** The 3D images were collected via the 3dMD cameras system (3dMD Corp). The raw data are triangle mesh with ~60,000 vertices and ~100,000 triangular faces.
2. **Data alignment I:** All raw 3D data were first aligned to the same position and orientation, where the subject's head and face pointed towards the y-axis and z-axis respectively. This was implemented by MATLAB function ’Rigid ICP Registration’ (Manu, 2022).
3. **Landmarking, facial cropping and template setting:** The 3D coordinates of landmarks were calculated, and used to crop the facial regions from the raw data. Firstly, we did the cropping on 200 random subjects. Then a template was made by averaging these 200 samples.
4. **Down-sampling for the template:** The template had about 20,000 vertices and 40,000 triangular faces. We used the open-source ACVD (Valette et al., 2008) package to down-sample the template into 5,023 vertices and 9,851 triangular faces. The down-sampled template maintained the essential morphology while having a slight size.
5. **Landmark-guide dense correspondence:** In this step, we deformed the template by rigid and non-rigid registration algorithm (Amberg et al., 2007), in order to approximate the aligned images from step 2).
6. **Data alignment II:** We aligned all template-based results from step 5), and produced the final data set.

**2. Implementation details about 3D graph convolutional networks**

***Implementation details***

The 3D graph convolutional networks is an extension of 2D convolution networks. This extension allows deep learning networks to cope with 3D graph data. In our study, we used the autoencoder developed by Gong et al., (2019), and we kept the default configurations except for setting the latent size to be 200. We trained the networks on 9,017 data points of both 9-year-old and 13-year-old data, and stopped the training after 600 epochs. The training was based on self-supervised, and no labels were involved. After training, the parameters of the networks, together with the 200 latent traits of each facial shape, were saved as files.

***Reconstruction quality***

Higher reconstruction quality means better low-dimension representation of 3D input in the latent features. We computed a heatmap to display the reconstruction error. We also did experiments on different latent sizes, in order to understand how the latent size influenced the reconstruction quality. Then, we made a trade-off between reconstruction error and dimensional complexity and set the latent size as 200 in this study.


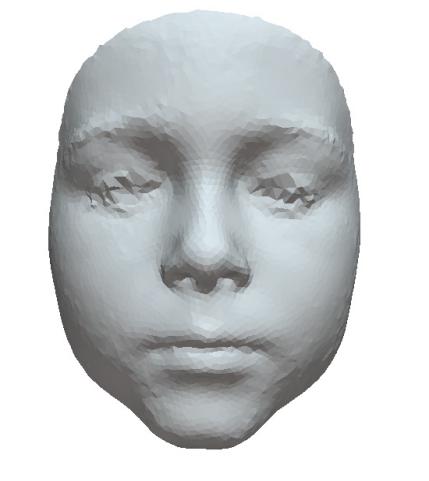

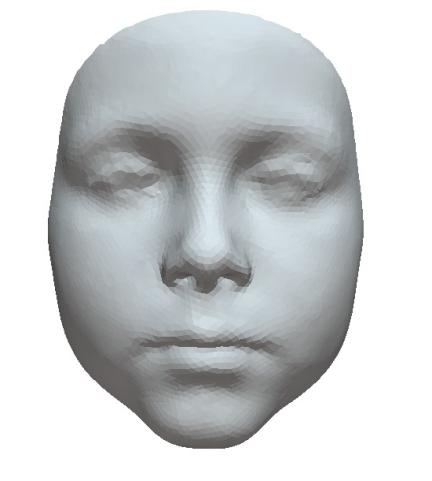

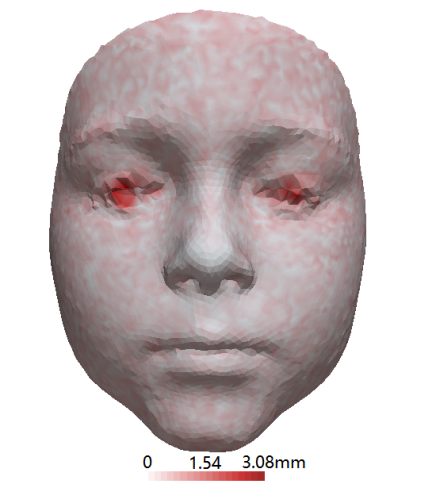


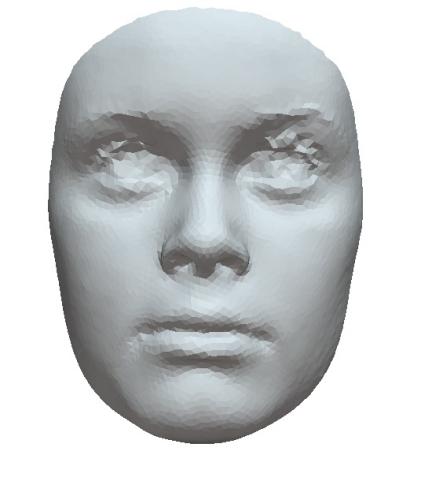

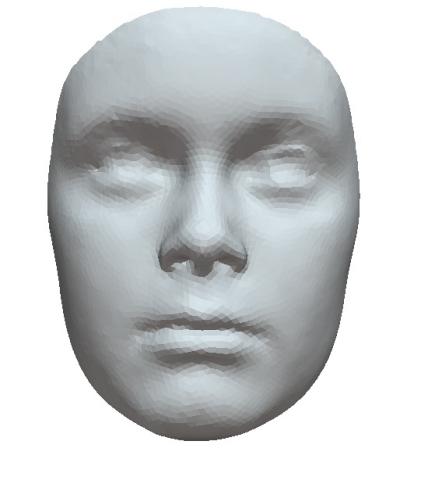

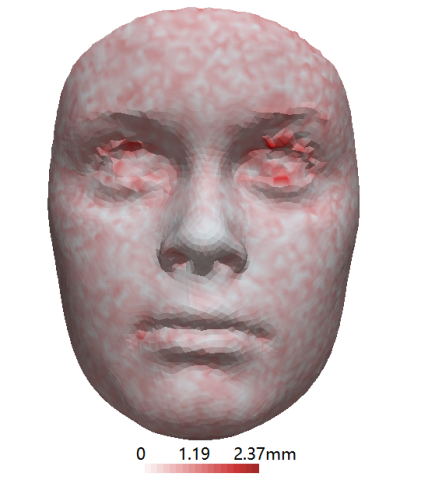


**Figure S1**: Reconstruction quality. left: input face; middle: reconstructed face; right: reconstruction error.

**Figure S2:** Reconstruction error of different latent dimensions.

***Interpretation of 200 facial traits***

After training the networks, the 200 traits are generated by the encoder, and each of them represents different structures on the 3D face. To better understand what each trait is representing, in the decoding stage, we changed the value of one single trait and observed the corresponding facial changes (**Figure S3a**). Furthermore, since each trait may be corresponding to different amounts of facial changes, there is unfairness between each trait. To address this, we defined a uniform value, $f(z)$, to measure the effect size of each trait on the face:

$$f\left( z_{i} \right)=Sum\_Dist( Decode\left( \left[ \mu_{0}, \mu_{1}, \ldots, \mu_{i},\ldots, \mu_{199} \right] \right), Decode\left( \left[ \mu_{0}, \mu_{1}, \ldots, \mu_{i}+\sigma_{i},\ldots, \mu_{199} \right] \right) ) (Equation S1)$$

where $\mu_{i}$ and $\sigma_{i}$ refer to the mean and standard deviation of $z_{i}$($N=N_{sample}$) respectively, and $Sum\_Dist()$ is defined as the sum of Euclidean distance between all paired points from two 3D faces.

As shown in **Figure S3**, the interpretation is displayed by heatmaps, where red areas refer to inward facial changes to the reference point, while blue areas refer to outward changes with respect to the reference point.


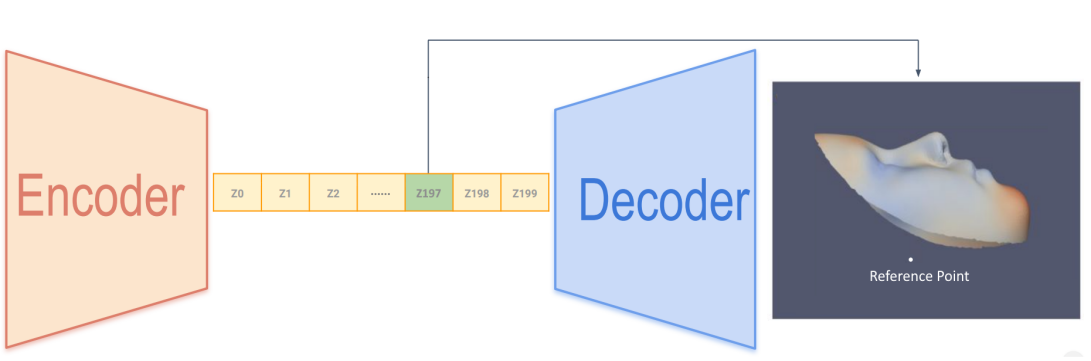


(a) Facial representation of one single trait


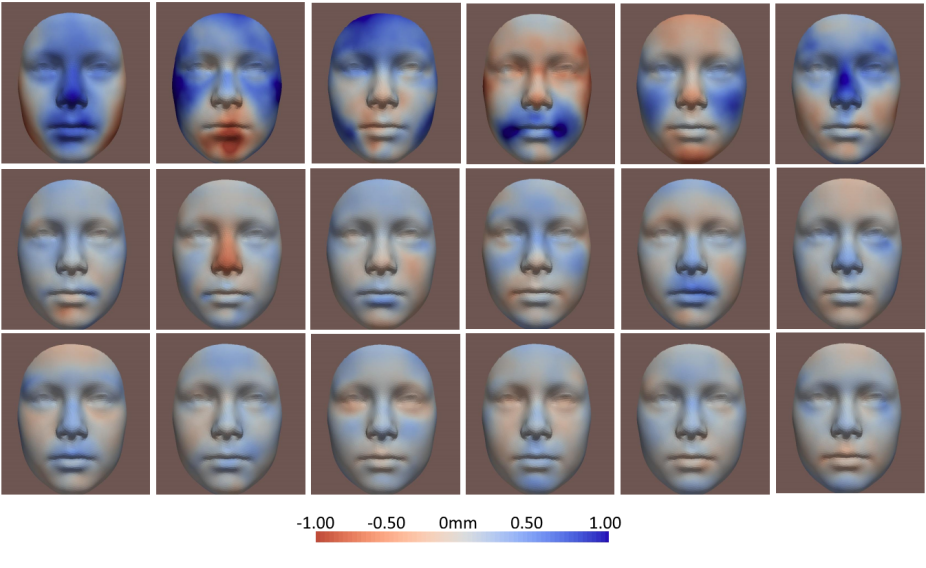


(b) Interpretation of each trait

**Figure S3:** Interpretation of 200 traits. **a)** Facial changes corresponding to one trait; **b**) Interpretation of each single trait, sorted from major to minor facial changes based on the values of f(z).

***Mapping selected traits back to the 3D face***

After selecting traits with statistical significance, we computed their corresponding effects on the 3D face. This process is similar with **Figure S3a**, but we computed the corresponding effects from multiple traits rather than single traits. As discussed, we performed Linear Regression on each trait $z_{i}$:

$z_{i}= \beta_{i0}+\beta_{i1}x_{PAE}+\beta_{i2}x_{Materal\_age}+\beta_{i3}x_{Materal\_smoking}+\beta_{i4}x_{Children\_age}+\beta_{i5}x_{Children\_BMI}+\beta_{i6}x_{Chidlren\_gender}+\beta_{i7}x_{Children\_ethnicity} , i=0,1,2\ldots,199 (Equation S2)$

**Equation S2** indicates that, the difference between non-exposed and exposed faces is corresponding to a change of $\beta_{i1}$ on $z_{i}$. Base on this, we combined all significant traits by setting $\beta_{i1}$ as their weights. As shown in **Figure S4**, in the decoding stage, we changed the values of all selected traits at the same time, and thus map these traits back to the 3D face:

$$\boldsymbol{F}_{\boldsymbol{baseline}}=Decode\left( \left[ \mu_{0}, \mu_{1},\mu_{2}, \ldots, \mu_{i},\ldots, \mu_{199} \right] \right) (Equation S3)$$

$$\boldsymbol{F}_{\boldsymbol{changed}}=Decode\left( \left[ \mu_{0}, \mu_{1}+\beta_{11},\mu_{2}+\beta_{21}, \ldots, \mu_{i}+\beta_{i1},\ldots, \mu_{199} \right] \right) (Equation S4)$$

$$Heatmap=PDist(\boldsymbol{F}_{\boldsymbol{change}}, ref\_point)-PDist\left( \boldsymbol{F}_{\boldsymbol{baseline}}\boldsymbol{,} ref_{point} \right) (Equation S5)$$

where $\mu_{i}$ refers to the mean of $z_{i}$($N=N_{sample}$), $\beta_{11}$, $\beta_{21}$ and $\beta_{i1}$ are coefficients from the regression results, $\boldsymbol{F}_{\boldsymbol{baseline}}$ is the average 3D face while $\boldsymbol{F}_{\boldsymbol{change}}$ is the face affected by selected traits, $PDist()$ is defined as the point-wise Euclidean distance between each point on the 3D face and the reference point.


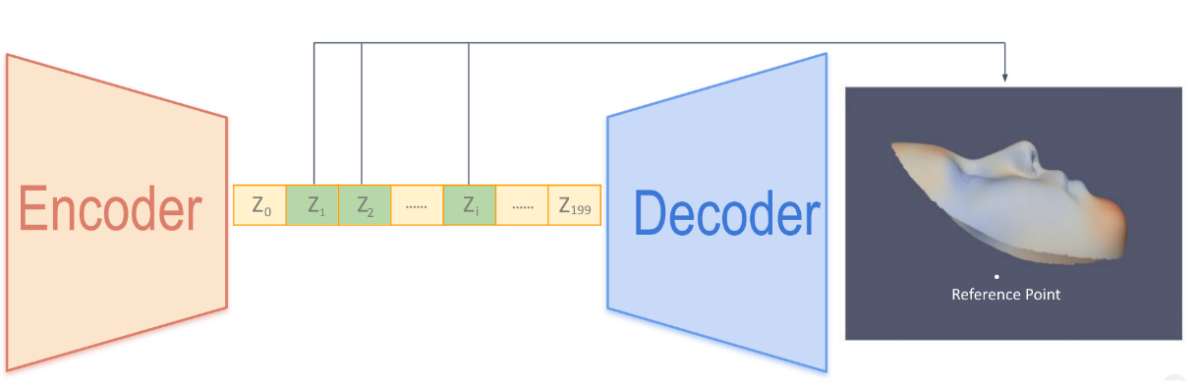


**Figure S4:** Combination of selected traits.

**3. Details about different levels of prenatal alcohol exposure (PAE) in the study population**

**Table SI:** Details about different levels of maternal alcohol consumption.

| **characteristic** | **9-year-old** | | **13-year-old** | |
| --- | --- | --- | --- | --- |
|  | **Non-exposed**  **(Control)** | **Any**  **Alcohol** | **Non-exposed**  **(Control)** | **Any**  **Alcohol** |
| Levels of PAE (%) |  |  |  |  |
| PAE Tier 1 |  |  |  |  |
| Drank alcohol before pregnancy only, level = 1 | 760 | 428 (60.6) | 519 | 349 (60.3) |
| Drank alcohol before pregnancy only, level = 2 | 760 | 158 (22.4) | 519 | 134 (23.1) |
| Drank alcohol before pregnancy only, level = 3 | 760 | 78 (11.0) | 519 | 56 (9.7) |
| Drank alcohol before pregnancy only, level = 4 | 760 | 28 (4.0) | 519 | 23 (4.0) |
| Drank alcohol before pregnancy only, level = 5 | 760 | 11 (1.6) | 519 | 15 (2.6) |
| Drank alcohol before pregnancy only, level = 6 | 760 | 3 (0.4) | 519 | 2 (0.3) |
| PAE Tier 2a |  |  |  |  |
| During pregnancy, any alcohol, level = 1 | 760 | 445(44.1) | 519 | 360(43.8) |
| During pregnancy, any alcohol, level = 2 | 760 | 354(35.1) | 519 | 286(34.8) |
| During pregnancy, any alcohol, level = 3 | 760 | 111(11.0) | 519 | 103(12.5) |
| During pregnancy, any alcohol, level = 4 | 760 | 46(4.6) | 519 | 27(3.3) |
| During pregnancy, any alcohol, level = 5 | 760 | 47(4.7) | 519 | 43(5.2) |
| During pregnancy, any alcohol, level = 6 | 760 | 5(0.5) | 519 | 3(0.4) |
| PAE Tier 2b |  |  |  |  |
| During pregnancy, any alcohol, level = 1 | 760 | 927(55.1) | 519 | 759 (55.0) |
| During pregnancy, any alcohol, level = 2 | 760 | 518(30.8) | 519 | 419 (30.4) |
| During pregnancy, any alcohol, level = 3 | 760 | 125(7.4) | 519 | 118 (8.6) |
| During pregnancy, any alcohol, level = 4 | 760 | 58(3.4) | 519 | 36 (2.6) |
| During pregnancy, any alcohol, level = 5 | 760 | 50(3.0) | 519 | 44 (3.2) |
| During pregnancy, any alcohol, level = 6 | 760 | 5(0.3) | 519 | 3 (0.2) |

Level1: <1 drink per week;

Level2: 1–3 per week;

Level3: 4–6 per week;

Level4: 1 per day;

Level5: 2–3 per day;

Level6: >3 per day.

An average alcoholic drink contains about 12 g of alcohol.

**4. Details about dose-response assessment**

**Table SII:** FDR-significant traits of different levels of PAE, Tier 2b, multi-ethnic for 9-year-old children.

| **Traits** i**ndex** | **p-value** | **FDR-corrected p-value** | **coefficient** | **Standard error** | **Mean** | **Standard deviation** | **f(z)** |
| --- | --- | --- | --- | --- | --- | --- | --- |
| **PAE level 1. Ne = 887, Nc = 760.** | | | | | | | |
| **125** | 9.7e-05 | 0.019 | 0.016 | 0.0040 | 0.016 | 0.066 | 287.89 |
| **51** | 1.0e-04 | 0.010 | -0.029 | 0.0075 | -0.002 | 0.125 | 833.34 |
| **173** | 6.1e-04 | 0.041 | -0.011 | 0.0032 | -0.006 | 0.050 | 185.23 |
| **PAE level 2-3. Ne = 546, Nc = 760.** | | | | | | | |
| **139** | 8.1e-05 | 0.016 | -0.013 | 0.0034 | 0.007 | 0.048 | 189.49 |
| **51** | 1.2e-04 | 0.012 | -0.033 | 0.0085 | -0.002 | 0.125 | 833.34 |
| **36** | 6.1e-04 | 0.041 | 0.016 | 0.0045 | 0.010 | 0.064 | 269.78 |
| **29** | 9.4e-04 | 0.047 | 0.011 | 0.0034 | 0.008 | 0.052 | 197.95 |
| **PAE level 4-6. Ne = 79, Nc = 760.** | | | | | | | |
| **173** | 3.7e-05 | 0.0073 | -0.026 | 0.0063 | -0.006 | 0.050 | 185.23 |
| **157** | 2.5e-04 | 0.025 | -0.026 | 0.0070 | 0.003 | 0.054 | 220.76 |
| **36** | 8.7e-04 | 0.027 | 0.030 | 0.0084 | 0.010 | 0.064 | 269.78 |
| **29** | 8.7e-04 | 0.044 | 0.021 | 0.0063 | 0.008 | 0.052 | 197.95 |
| **69** | 1.1e-03 | 0.046 | 0.035 | 0.011 | -0.017 | 0.080 | 485.34 |

Ne refers to the number of the exposed samples, while Nc refers to the number of the control samples.

**5. Details about nominal significant traits**

**Table SIII:** Details about nominal significant traits, Tier 2b, PAE level > 1, multi-ethnic.

| **Traits** i**ndex** | **p-value** | **FDR-corrected p-value** | **coefficient** | **SE** | **Mean** | **Std** | **f(z)** |
| --- | --- | --- | --- | --- | --- | --- | --- |
| **9-year-old children. Ne = 756, Nc = 760** | | | | | | | |
| **36*** | 7.1e-05 | 0.014 | 0.017 | 0.0044 | 0.010 | 0.064 | 269.78 |
| **139*** | 9.3e-05 | 0.009 | -0.013 | 0.0033 | 0.007 | 0.048 | 189.49 |
| **29*** | 0.0002 | 0.013 | 0.013 | 0.0034 | 0.008 | 0.052 | 197.95 |
| **51*** | 0.0002 | 0.012 | -0.030 | 0.0083 | -0.002 | 0.125 | 833.34 |
| **69*** | 0.0006 | 0.023 | 0.019 | 0.0055 | -0.017 | 0.080 | 485.34 |
| **173*** | 0.0009 | 0.030 | -0.011 | 0.0033 | -0.006 | 0.050 | 185.23 |
| **87*** | 0.001 | 0.036 | -0.010 | 0.0031 | 0.000 | 0.044 | 155.85 |
| **57*** | 0.002 | 0.048 | 0.022 | 0.0070 | -0.013 | 0.101 | 648.42 |
| **125** | 0.005 | 0.101 | 0.013 | 0.0045 | 0.016 | 0.066 | 287.89 |
| **12** | 0.005 | 0.092 | -0.037 | 0.0129 | 0.070 | 0.208 | 1852.95 |
| **178** | 0.006 | 0.102 | 0.010 | 0.0036 | 0.000 | 0.054 | 209.58 |
| **58** | 0.006 | 0.107 | 0.011 | 0.0041 | 0.007 | 0.059 | 255.12 |
| **174** | 0.007 | 0.106 | 0.016 | 0.0058 | 0.040 | 0.092 | 493.61 |
| **163** | 0.009 | 0.132 | 0.010 | 0.0038 | -0.002 | 0.054 | 214.16 |
| **97** | 0.010 | 0.135 | -0.015 | 0.0058 | 0.012 | 0.082 | 419.65 |
| **55** | 0.011 | 0.135 | -0.012 | 0.0048 | 0.005 | 0.068 | 285.65 |
| **75** | 0.012 | 0.141 | 0.008 | 0.0033 | -0.006 | 0.048 | 181.64 |
| **121** | 0.018 | 0.198 | -0.009 | 0.0037 | -0.007 | 0.057 | 227.44 |
| **83** | 0.018 | 0.188 | -0.009 | 0.0036 | 0.006 | 0.053 | 190.46 |
| **39** | 0.018 | 0.180 | 0.012 | 0.0049 | -0.002 | 0.072 | 345.68 |
| **54** | 0.024 | 0.224 | 0.008 | 0.0036 | -0.008 | 0.053 | 221.60 |
| **53** | 0.026 | 0.239 | 0.007 | 0.0032 | 0.019 | 0.047 | 177.26 |
| **19** | 0.035 | 0.305 | 0.012 | 0.0055 | -0.012 | 0.086 | 484.90 |
| **47** | 0.040 | 0.330 | -0.007 | 0.0032 | 0.004 | 0.047 | 178.17 |
| **160** | 0.042 | 0.332 | -0.008 | 0.0037 | -0.008 | 0.053 | 203.75 |
| **185** | 0.042 | 0.320 | -0.007 | 0.0033 | 0.002 | 0.049 | 172.72 |
| **7** | 0.042 | 0.314 | -0.007 | 0.0036 | 0.005 | 0.053 | 214.33 |
| **128** | 0.046 | 0.330 | -0.006 | 0.0031 | -0.003 | 0.046 | 148.50 |
| **13-year-old children. Ne = 620, Nc = 519** | | | | | | | |
| **74** | 0.0004 | 0.071 | 0.018 | 0.0050 | -0.011 | 0.066 | 269.42 |
| **12** | 0.0006 | 0.064 | -0.061 | 0.0179 | -0.028 | 0.245 | 2185.73 |
| **45** | 0.003 | 0.182 | 0.013 | 0.0045 | 0.009 | 0.057 | 213.14 |
| **193** | 0.005 | 0.239 | 0.018 | 0.0064 | -0.018 | 0.084 | 414.69 |
| **85** | 0.007 | 0.272 | -0.011 | 0.0040 | 0.001 | 0.052 | 176.85 |
| **164** | 0.007 | 0.247 | -0.012 | 0.0043 | 0.001 | 0.054 | 204.62 |
| **14** | 0.011 | 0.303 | 0.031 | 0.0122 | 0.013 | 0.158 | 1126.57 |
| **46** | 0.012 | 0.289 | 0.009 | 0.0037 | 0.008 | 0.048 | 176.55 |
| **90** | 0.012 | 0.258 | -0.014 | 0.0055 | 0.013 | 0.072 | 343.16 |
| **172** | 0.012 | 0.237 | -0.010 | 0.0038 | 0.002 | 0.050 | 170.99 |
| **26** | 0.012 | 0.218 | -0.011 | 0.0043 | -0.001 | 0.056 | 226.21 |
| **5** | 0.013 | 0.219 | -0.011 | 0.0044 | 0.015 | 0.055 | 248.72 |
| **196** | 0.015 | 0.234 | -0.021 | 0.0088 | 0.008 | 0.116 | 674.34 |
| **16** | 0.017 | 0.242 | -0.013 | 0.0053 | -0.007 | 0.067 | 308.31 |
| **66** | 0.017 | 0.227 | 0.009 | 0.0036 | 0.003 | 0.049 | 180.30 |
| **51** | 0.024 | 0.296 | -0.022 | 0.0098 | 0.034 | 0.135 | 898.73 |
| **197** | 0.025 | 0.290 | -0.008 | 0.0037 | -0.009 | 0.047 | 168.62 |
| **7** | 0.027 | 0.296 | -0.009 | 0.0040 | -0.003 | 0.051 | 207.37 |
| **87** | 0.034 | 0.357 | -0.008 | 0.0039 | 0.001 | 0.050 | 176.29 |
| **75** | 0.035 | 0.351 | 0.008 | 0.0036 | -0.006 | 0.047 | 179.60 |
| **109** | 0.043 | 0.405 | -0.008 | 0.0041 | -0.006 | 0.053 | 223.21 |
| **80** | 0.043 | 0.392 | 0.009 | 0.0043 | -0.004 | 0.054 | 211.15 |
| **96** | 0.045 | 0.391 | -0.011 | 0.0054 | 0.018 | 0.072 | 325.94 |
| **47** | 0.049 | 0.407 | -0.007 | 0.0037 | -0.002 | 0.048 | 179.54 |
| **‘growth’. Ne = 460, Nc = 377.** | | | | | | | |
| **12** | 0.017 | 0.236 | -0.040 | 0.0168 | 0.026 | 0.231 | 2053.41 |
| **16** | 0.008 | 0.761 | -0.014 | 0.0052 | 0.005 | 0.065 | 299.80 |
| **19** | 0.022 | 0.850 | -0.018 | 0.0079 | -0.010 | 0.094 | 531.21 |
| **36** | 0.013 | 0.702 | -0.015 | 0.0061 | 0.012 | 0.063 | 267.30 |
| **43** | 0.028 | 0.591 | 0.009 | 0.0042 | 0.002 | 0.045 | 162.83 |
| **53** | 0.001 | 0.544 | -0.015 | 0.0046 | 0.012 | 0.047 | 175.87 |
| **66** | 0.028 | 0.472 | 0.010 | 0.0046 | 0.001 | 0.048 | 177.34 |
| **74** | 0.037 | 0.430 | 0.011 | 0.0054 | -0.005 | 0.064 | 261.61 |
| **81** | 0.023 | 0.479 | -0.021 | 0.0093 | -0.004 | 0.094 | 527.73 |
| **93** | 0.036 | 0.463 | 0.010 | 0.0050 | -0.002 | 0.050 | 187.42 |
| **97** | 0.015 | 0.505 | 0.018 | 0.0072 | 0.017 | 0.085 | 431.61 |
| **139** | 0.014 | 0.469 | 0.011 | 0.0044 | 0.005 | 0.049 | 194.45 |
| **156** | 0.044 | 0.526 | 0.010 | 0.0048 | 0.000 | 0.049 | 185.81 |
| **164** | 0.017 | 0.510 | -0.012 | 0.0050 | -0.002 | 0.054 | 203.93 |
| **172** | 0.016 | 0.491 | -0.011 | 0.0046 | 0.003 | 0.049 | 169.76 |
| **183** | 0.034 | 0.545 | 0.013 | 0.0062 | 0.004 | 0.062 | 270.37 |

Ne refers to the number of the exposed samples, while Nc refers to the number of the control samples.

As defined in Equation S1, f(z) is the effect size of the trait on the facial shape.

Index with * was still significant after correction for multiple testing with FDR.

**6. Detailed visualization results**


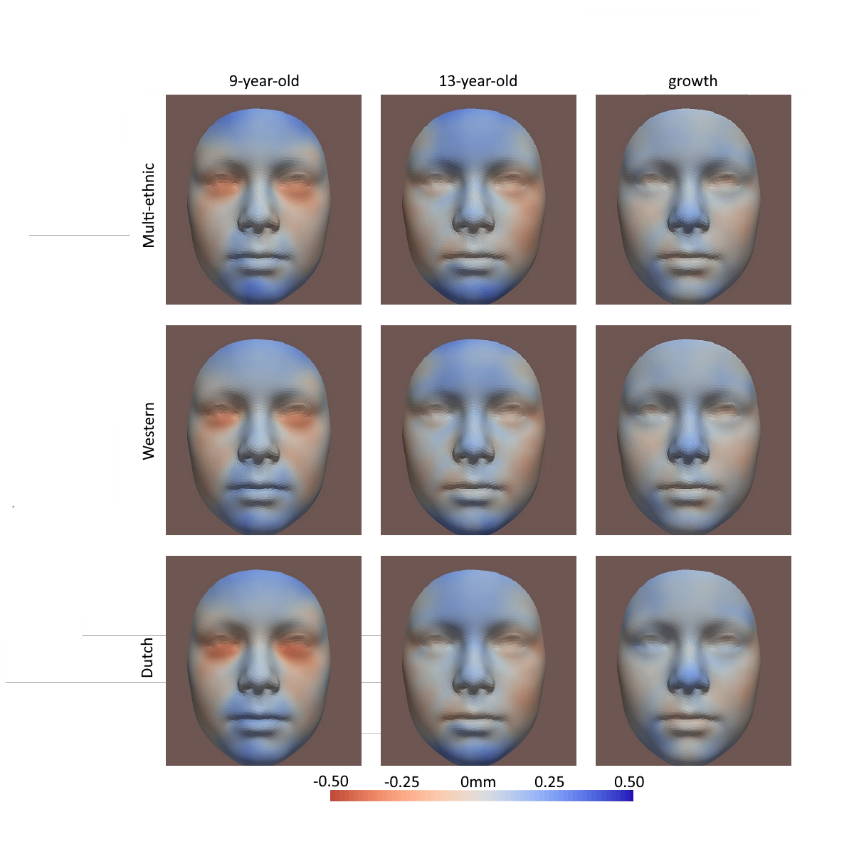


**Figure S5:** Nominal significant results (p-value<0.05). Tier 2b, PAE level > 1.

(a) Tier 2a (b) Tier 2b

**Figure S6:** Nominal significant results of different levels of PAE, in multi-ethnic group.

**7. Detailed PAE prediction results**

**Table SIV:** Sorted odds ratio of top 8 traits.

Ne refers to the number of the exposed samples, while Nc refers to the number of the control samples.

| **9-year-old**  **(Ne = 670, Nc = 329)** | | | **13-year-old**  **(Ne = 543, Nc = 236)** | | |
| --- | --- | --- | --- | --- | --- |
| **Trait** **index** | **odds ratio** | **p-value** | **Trait index** | **odds ratio** | **p-value** |
| **36** | 1.25 | 0.0077 | **14** | 1.36 | 0.0039 |
| **57** | 1.16 | 0.065 | **74** | 1.33 | 0.0060 |
| **39** | 1.15 | 0.11 | **45** | 1.28 | 0.013 |
| **125** | 1.15 | 0.10 | **96** | 0.88 | 0.18 |
| **69** | 1.13 | 0.16 | **12** | 0.86 | 0.15 |
| **139** | 0.87 | 0.10 | **85** | 0.86 | 0.11 |
| **83** | 0.82 | 0.016 | **87** | 0.81 | 0.027 |
| **87** | 0.76 | 0.00083 | **164** | 0.76 | 0.0069 |

**Table SV:** Sorted *F*-test results of top 8 traits.

| **9-year-old**  **(Ne = 670, Nc = 329)** | | | **13-year-old**  **(Ne = 543, Nc = 236)** | | |
| --- | --- | --- | --- | --- | --- |
| **Trait index** | **p-value** | **F-value** | **Trait index** | **p-value** | **F-value** |
| **39** | 7.6e-6 | 20.3 | **12** | 3.5e-7 | 26.4 |
| **139** | 9.7e-5 | 15.3 | **164** | 1.8e-4 | 14.2 |
| **87** | 1.5e-4 | 14.5 | **74** | 1.8e-4 | 14.1 |
| **36** | 3.4e-4 | 12.9 | **85** | 3.8e-4 | 12.7 |
| **57** | 5.3e-4 | 12.1 | **96** | 9.6e-3 | 6.7 |
| **125** | 2.1e-3 | 9.5 | **14** | 1.6e-2 | 5.9 |
| **69** | 1.1e-2 | 6.4 | **87** | 1.6e-2 | 5.8 |
| **83** | 0.077 | 3.1 | **45** | 0.29 | 1.1 |

**8. Visualize the difference between sex groups**

**Table SVI:** Top 5 FDR-significant facial traits associated with sex.

| **Traits** i**ndex** | **p-value** | **FDR-corrected p-value** | **coefficient** | **Standard error** |
| --- | --- | --- | --- | --- |
| **158** | 5.7e-31 | 1.1e-28 | -0.062 | 0.0070 |
| **181** | 1.8e-24 | 1.8e-22 | 0.057 | 0.0074 |
| **32** | 4.8e-21 | 3.2e-19 | 0.029 | 0.0040 |
| **71** | 5.4e-18 | 2.7e-16 | -0.062 | 0.0094 |
| **12** | 4.18e-17 | 7.14e-16 | 0.084 | 0.0129 |
| …  n=129 traits in total survived FDR | | | | |


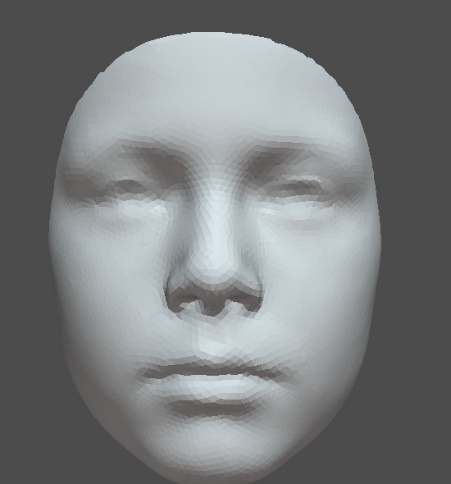

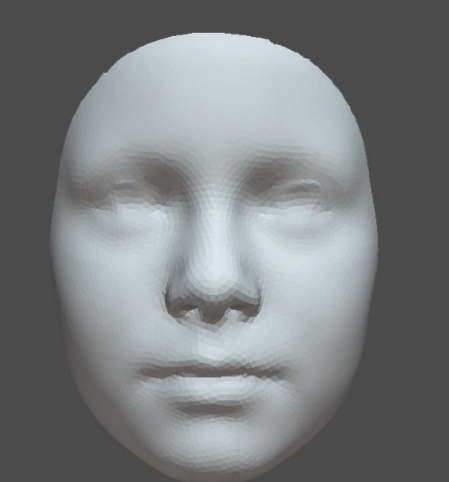

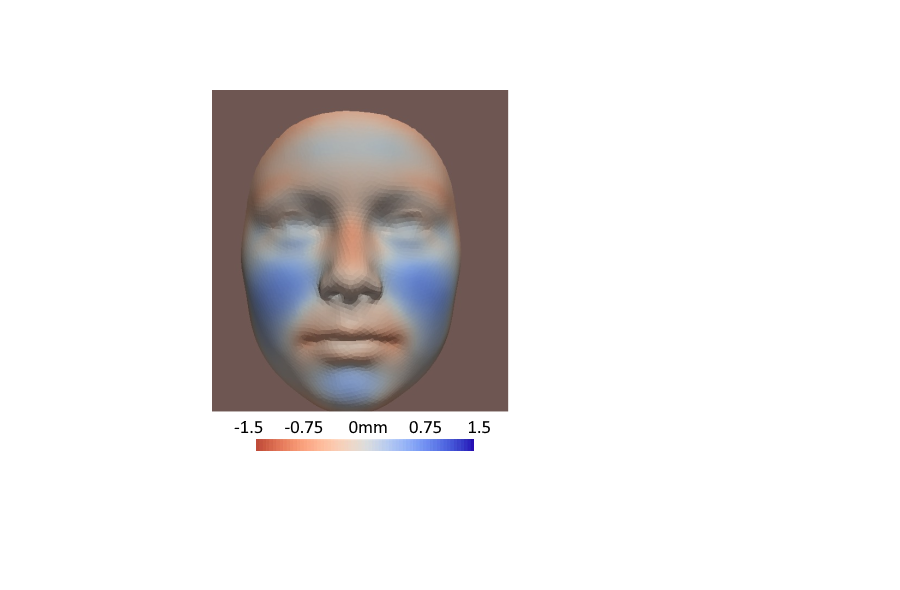


(a) (b) (c)

**Figure S7:** Facial different of sex. a) Average of boys; b) average of girls; c) facial changes from boys to girls

**9. Correlations between latent dimensions**

Here we show the correlations between latent dimensions. Only the histogram shows both positive and negative correlations, while all the correlation matrix show the absolute value of correlations.

**
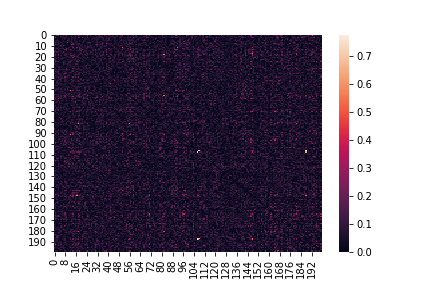
**

(a) (b)

**Figure S8:** The overall correlations between 200 latent dimensions. **a)** Correlation matrix; **b)** Histogram of correlations.

**
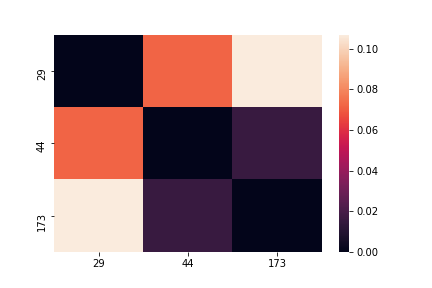

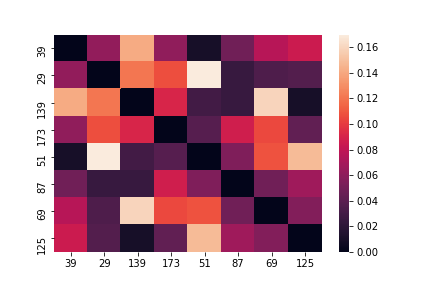

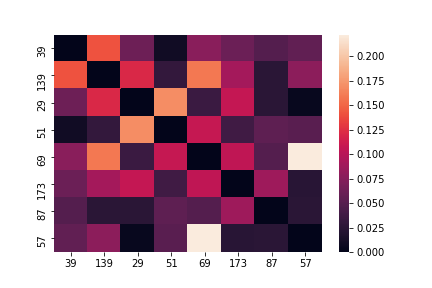
**

Tier 1 Tier 2a Tier 2b

**Figure S9:** The correlations between FDR-significant traits of different tiers, corresponding to the traits from **Table II.**

**
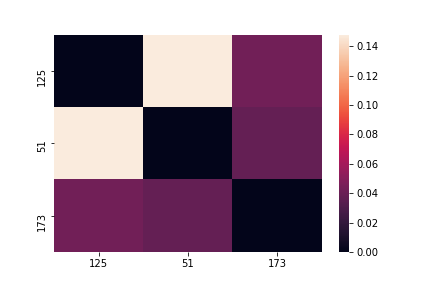

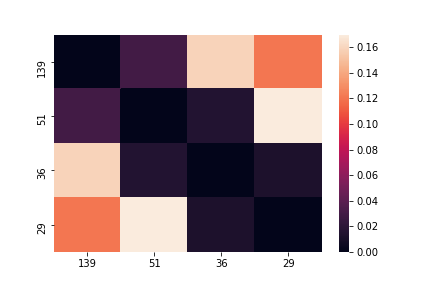

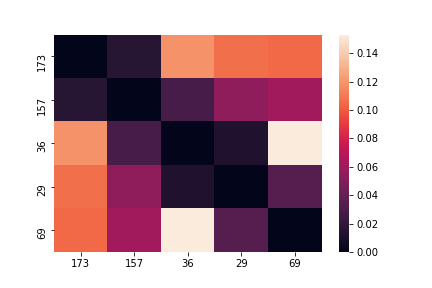
**

PAE level 1 PAE level 2-3 PAE level 4-6

**Figure S10:** The correlations between FDR-significant traits of different PAE levels, corresponding to the traits from **Table SII.**

**10. Replicability of the auto-encoder.**

In order to test the replicability of our results, we performed 3 additionally independent runs for the training of the auto-encoder, and then we performed linear regression analysis. These 3 runs followed the same experiment settings (i.e., PAE level, age, etc.) as Tier 2b in **Table II**. **Table SVII** shows the results of linear regression analysis, and **Figure S11** shows the visualization results.

**Table SVII:** FDR-significant traits for 9-year-old children, multi-ethnic, Tier 2b, PAE level >1, Ne = 756, Nc = 760.

| **Traits** i**ndex** | **p-value** | **FDR-corrected p-value** | **coefficient** | **SE** |
| --- | --- | --- | --- | --- |
| **Run #1** | | | | |
| **29** | 5.9e-06 | 0.001 | 0.015 | 0.0034 |
| **36** | 1.9e-04 | 0.019 | 0.015 | 0.0039 |
| **51** | 3.4e-04 | 0.023 | -0.031 | 0.0088 |
| **69** | 7.3e-04 | 0.036 | 0.019 | 0.0056 |
| **Run #2** | | | | |
| **29** | 7.0e-06 | 0.001 | 0.015 | 0.0034 |
| **36** | 2.0e-04 | 0.020 | 0.015 | 0.0040 |
| **51** | 4.2e-04 | 0.028 | -0.030 | 0.0085 |
| **Run #3** | | | | |
| **29** | 2.6e-06 | 0.001 | 0.016 | 0.0033 |
| **51** | 2.8e-04 | 0.028 | -0.031 | 0.0085 |
| **36** | 4.4e-04 | 0.029 | 0.014 | 0.0039 |
| **125** | 1.4e-03 | 0.046 | 0.014 | 0.0044 |
| **121** | 1.2e-03 | 0.039 | -0.013 | 0.0041 |


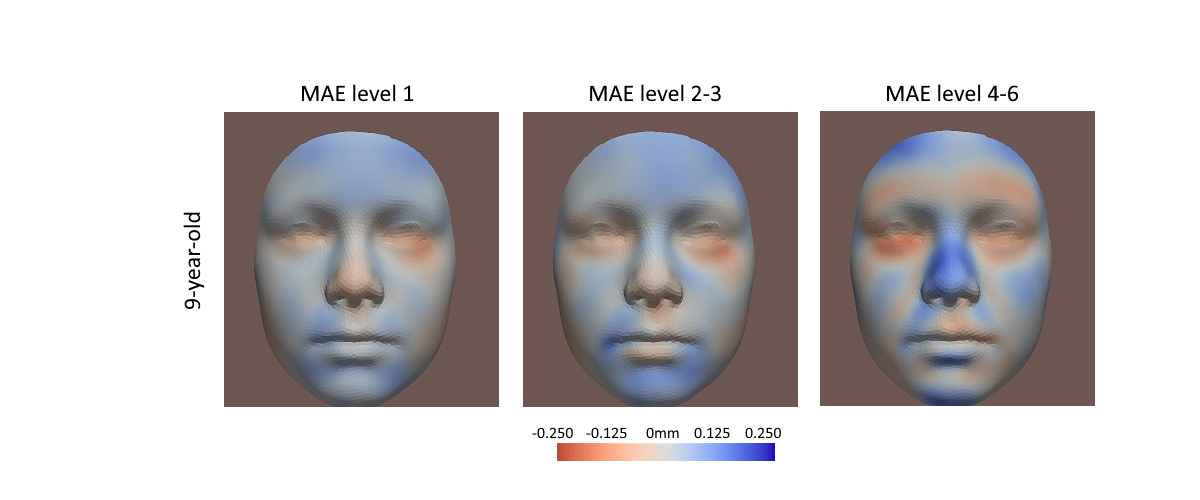


**
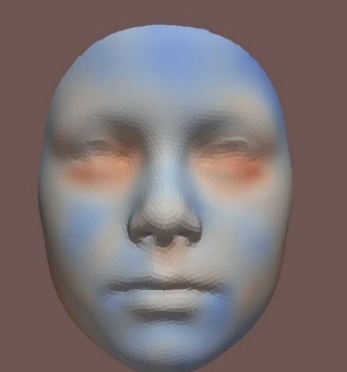

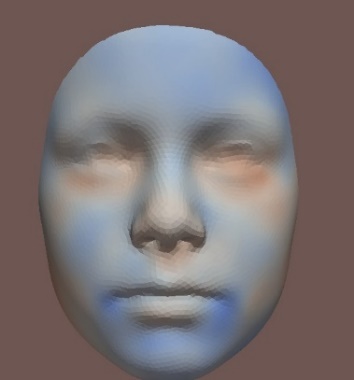

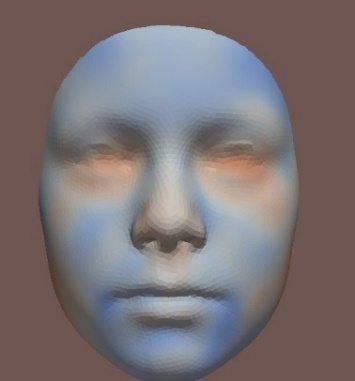
**

(a) (b) (c)

**Figure S11:** Visualization of FDR-significant traits. a) Run #1; b) Run#2; c) Run #3.

**11. A test of the multivariate approach.**

As suggested, we tested multivariate approach on the 200 facial traits. The multivariate approach used is the canonical correlation analysis (CCA). Linear regression was run on each trait for covariate control, and only residuals were input into the CCA. After CCA, the loadings were used as weights for the interpretation of residuals/traits. **Table SVIII** shows the loadings and **Figure S12** shows the visualization. The CCA test used the same settings (i.e., Tiers, age group, etc.) as Tier 2b in **Table II**. The CCA results were close to the trait-by-trait results: 1) The top traits from CCA (**Table SVIII**) are exactly traits that survived FDR in the trait-by-trait analysis (Tier 2b in **Table II**); 2) The facial heatmap derived from CCA is similar to our results.

**Table SVIII:** CCA loadings of 200 traits, sorted from high to low based on the absolute loading value,

in multi-ethnic 9-year-old children, Tier 2b, PAE level >1.

| **Traits** i**ndex** | **loadings** |
| --- | --- |
| **36** | 0.046 |
| **139** | -0.045 |
| **29** | 0.043 |
| **51** | -0.042 |
| **69** | 0.040 |
| **173** | -0.039 |
| **87** | -0.037 |
| **57** | 0.036 |
| **…** |  |
| **198** | -0.0005 |
| **8** | -0.0004 |
| **77** | -0.0004 |
| **100** | 0.0002 |


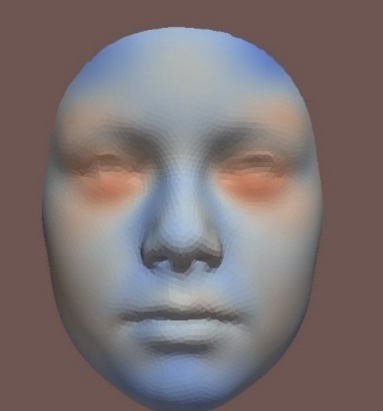


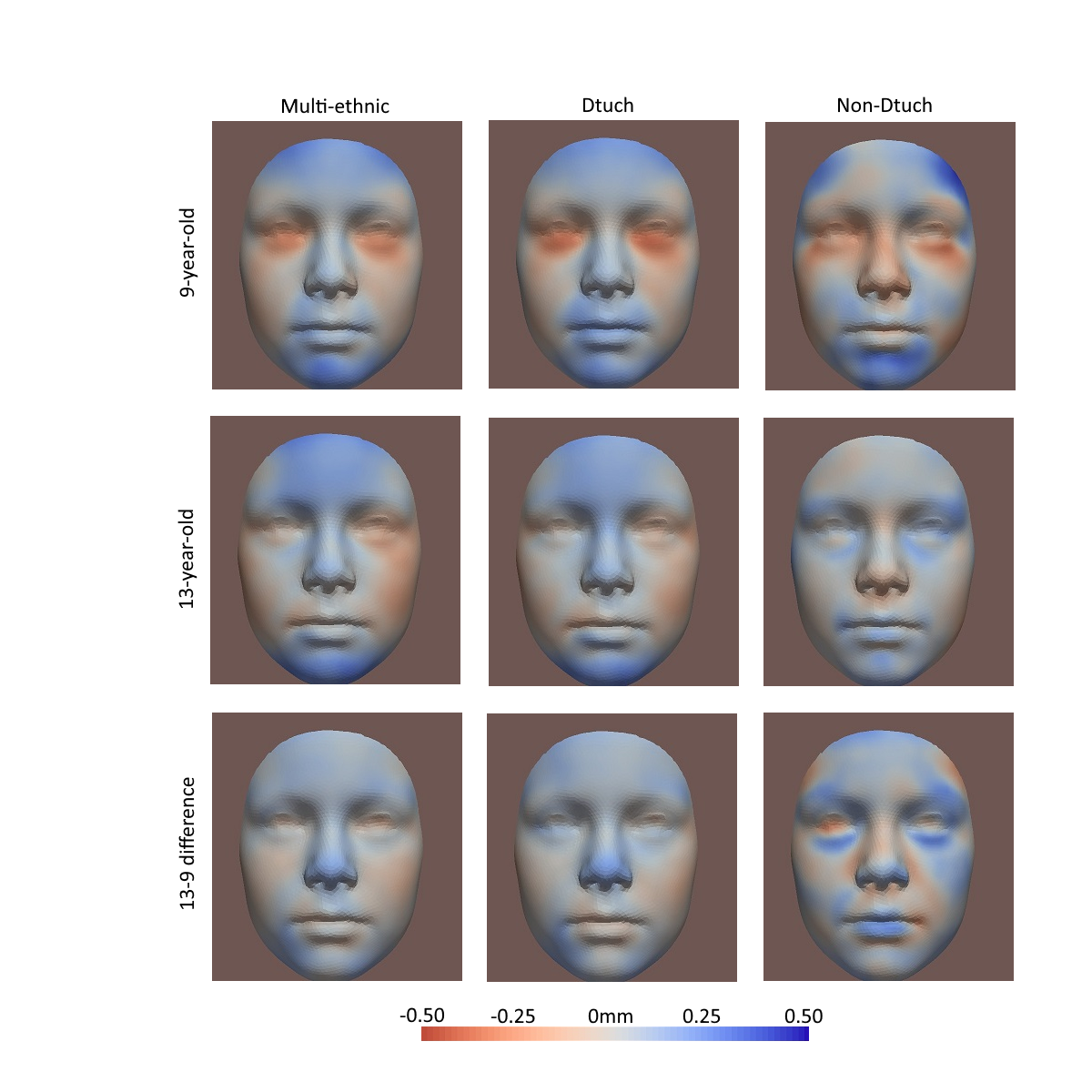


**Figure S12:** Visualization of the CCA results.

**12. Dutch-only Analysis compared with multi-ethnic analysis**

**Table SIX:** Top 10 significant traits from Dutch-only analysis and multi-ethnic analysis respectively,

In 9-year-old children, PAE level >1, Tier 2b.

| **Dutch-only**  **(Ne = 670, Nc = 329)** | | **Multi-ethnic**  **(Ne = 756, Nc = 760)** | |
| --- | --- | --- | --- |
| **Trait** **index** | **p-value** | **Trait** **index** | **p-value** |
| **87** | 2.93e-05 | **36** | 7.1e-05 |
| **36** | 7.03e-05 | **139** | 9.3e-05 |
| **69** | 1.68e-03 | **29** | 1.9e-04 |
| **57** | 2.12e-03 | **51** | 2.5e-04 |
| **139** | 3.33e-03 | **69** | 5.6e-04 |
| **83** | 6.27e-03 | **173** | 8.9e-04 |
| **125** | 6.41e-03 | **87** | 1.3e-03 |
| **39** | 7.55e-03 | **57** | 1.9e-03 |
| **173** | 7.80e-03 | **125** | 4.6e-03 |
| **51** | 1.07e-02 | **12** | 4.6e-03 |

Trait index was sorted based on p-value.

**13. Comparison between autoencoder (AE) and principal component analysis (PCA)**

As suggested, we compared the AE-based model to a PCA-based model in terms of their generalization and specificity, following settings from Nauwelaers et al. (2021). In the experiments, N = 9,017 facial scans were included, and results were calculated based on a 5-fold cross validation. As shown in **Table SX**, in all latent dimensions (64, 128 and 200), the AE-based model showed slightly better generalization than PCA. Moreover, the AE-based model also showed improved specificity in all latent dimensions, which indicates the presence of non-linearity in 3D facial shape data.

**Table SX:** Results of the comparison between AE and PCA.

| **Model** | **Generalization**  **Mean ± SD (mm)** | **Specificity**  **Mean ± SD (mm)** |
| --- | --- | --- |
| **Latent dimension: 64** | | |
| **PCA** | 0.265±0.00085 | 1.385±0.0027 |
| **AE** | 0.255±0.00067 | 1.328±0.0047 |
| **Latent dimension: 128** | | |
| **PCA** | 0.234±0.00042 | 1.408±0.0026 |
| **AE** | 0.229±0.00048 | 1.330±0.0071 |
| **Latent dimension: 200** | | |
| **PCA** | 0.223±0.00034 | 1.414±0.0030 |
| **AE** | 0.220±0.00038 | 1.348±0.0100 |
